# Does COVID‐19 Vaccination Protect Against Post‐COVID Cardiovascular Conditions?

**DOI:** 10.1002/rmv.70219

**Published:** 2026-10-10

**Authors:** Lidian Franci Batalha Santa Maria, Márcio Coutinho Xavier Naves, Elisabeth Carmen Duarte, Isis Polianna Silva Ferreira de Carvalho, Henry Maia Peixoto

**Affiliations:** ^1^ Faculty of Medicine Center of Tropical Medicine Campus Universitário Darcy Ribeiro University of Brasilia Brasília Brazil; ^2^ Cardiology and Transplant Institute of the Federal District Brasília Brazil; ^3^ Department of Science and Technology Secretariat of Science Technology, Innovation and Health Complex Ministry of Health Brasília Brazil

**Keywords:** cardiovascular diseases, causality, COVID‐19, COVID‐19 vaccines, post‐COVID conditions

## Abstract

Cardiovascular diseases, such as acute myocardial infarction, heart failure, and arrhythmias, have been associated with SARS‐CoV‐2 infection, representing an important cause of long‐term morbidity and mortality. In addition to preventing primary infection, COVID‐19 vaccination has emerged as a protective factor against post‐COVID cardiovascular outcomes. This study aims to analyse the evidence supporting a causal relationship between COVID‐19 vaccination and the prevention of post‐COVID cardiovascular conditions, based on classical and contemporary epidemiological criteria, including the Bradford Hill criteria (BHC) and the sufficient component cause (SCC) model. A qualitative synthesis of the evidence was performed according to the nine BHC and the SCC model. Scientific evidence indicates that COVID‐19 vaccination meets four of the nine BHC as a protective factor against post‐COVID cardiovascular conditions, namely strength of association, consistency, temporality, and biological gradient. Three criteria, biological plausibility, coherence, and analogy, were partially met. Specificity and experimental evidence were not supported by the available literature. The SCC model highlights the fundamental role of vaccination as a preventive causal component. It also contributes to two criteria, strength of association and temporality, while emphasising the limitation of specificity in causal inference. COVID‐19 vaccination has proven effective beyond the acute phase of the disease, with protection extending to a reduced risk of post‐COVID cardiovascular conditions. Comprehensive immunisation strategies and reinfection prevention are essential to mitigate the burden of post‐COVID conditions. Additional studies are needed to explore the underlying biological mechanisms, interactions, and the role of COVID‐19 vaccination in preventing post‐COVID complications.

## 1 Introduction

Post‐COVID condition (PCC) represents an important cause of long‐term morbidity [1] and mortality [2], even after the COVID‐19 pandemic. Evidence indicates that cardiovascular diseases such as acute myocardial infarction, heart failure, and arrhythmias are associated with prior SARS‐CoV‐2 infection [3] and constitute clinically relevant manifestations of PCC.

This association has been explained by both direct and indirect damage to the myocardium [4], directly through infection and indirectly through injuries triggered by the inflammatory response, endothelial dysfunction, and thrombus formation, among other mechanisms [5]. It is well established that, while the innate immune response is necessary to combat the virus, it can be harmful when excessive and persistent, potentially causing cellular damage [6].

Regarding PCC, there is an urgent need to expand the understanding of relevant risk and protective factors, particularly those associated with persistent cardiovascular conditions. In addition to preventing primary infection and severe and fatal cases, COVID‐19 vaccination has emerged as a protective factor against cardiovascular outcomes associated with PCC [7], both in individuals with preexisting cardiovascular disease and in those without such a history [8].

Beyond statistical association, although causal inference is a complex and challenging debate in epidemiology [9], it is essential for guiding public health policies and practices [10]. Some approaches have been used to inform discussions on causality, which, although not definitive on their own [9, 10, 11], provide a framework for understanding the natural history of diseases and identifying effective preventive measures.

## 2 Approaches to Disease Causality

According to the sufficient component cause (SCC) model of Rothman & Greenland (2005), the cause of a specific disease can be defined as a prior event, condition, or characteristic that was necessary for the disease to occur, assuming all other conditions are held constant [9]. A sufficient cause—that is, the complete causal mechanism‐is defined by the authors as a minimal set of conditions and events (component causes) that inevitably result in the disease [9]. In this model, multiple components interact, and when all required elements are present, they form a sufficient cause that inevitably leads to the occurrence of the disease. Each sufficient cause may include biological factors and specific effect mechanisms, highlighting the multifactorial nature of causal events [9].

Based on this theory, our hypothesis is that protective factors, such as COVID‐19 vaccination, may be part of causal mechanisms and reduce the likelihood of cardiovascular disease in the post‐COVID period. Vaccination could, for example, prevent or mitigate the effects of other underlying component causes, primarily the prior COVID‐19 infection itself, which is a necessary cause for the development of post‐COVID cardiovascular conditions [12].

In addition to the SCC model [9], the causality criteria proposed by Bradford Hill (BHC) also deserve attention [11]. The BHC is composed of nine viewpoints, which are presented and discussed sequentially throughout the manuscript.

Accordingly, the present study aims to analyse the evidence for a causal relationship between COVID‐19 vaccination and the prevention of post‐COVID cardiovascular conditions, based on both classical and contemporary epidemiological criteria, including the Bradford Hill criteria (BHC) [11] and the sufficient component cause (SCC) model [9].

## 3 Methods

The evidence on the protective effect of COVID‐19 vaccines against cardiovascular conditions in PCC was examined in detail, guided by the nine BHC [11] and the SCC model [9], based on the reviewed literature. The qualitative synthesis of the evidence included articles published between 2020 and 2025, from systematic reviews as well as other selected publications that made a meaningful contribution to assessing the hypothesis. The search was conducted in MEDLINE/PubMed, Embase, BVS, EBSCOhost, and the grey literature.

The authors used Claude (Claude Opus 5; Anthropic) to design, a conceptual vector diagram generated with Python/SVG from the manuscript content, and to check its consistency with the text. The authors reviewed and approved all content and take full responsibility for it.

## 4 Results and Discussion

A total of ten studies were selected to evaluate the nine Bradford Hill criteria. The publications included both original studies and meta‐analyses. Table 1 summarises the references used to support the evaluation of the Bradford Hill criteria.

**TABLE 1:** Analysis of bradford hill criteria (BHC) regarding COVID‐19 vaccination and protection against post‐COVID cardiovascular conditions, based on the reviewed literature.

Criteria/Outcome	Met	Partially met	Not met
1. Strength of association	X [13, 14]	—	—
2. Consistency	X [13, 14, 15]	—	—
3. Specificity	—	—	X
4. Temporality	X [13, 15, 16]	—	—
5. Biological gradient	X [13, 14, 16]	—	—
6. Plausibility	—	X [17, 18]	—
7. Coherence	—	X [19, 20]	—
8. Experimental evidence	—	—	X
9. Analogy	—	X [20, 21, 22]	—

### 4.1 Strength of the Association

Hill argued that strong associations are more likely to represent a causal relationship, whereas weak associations can more easily be explained by bias [11]. The SCC model reinforces the BHC's perspective on the strength of the association [10]. Within this framework, a causal component can play a decisive role in forming a sufficient cause, thereby increasing the likelihood that the effect will persist even in the presence of some bias.

When applying this criterion to the protective effect of the COVID‐19 vaccine against cardiovascular diseases, a study conducted in the United States examined cardiovascular outcomes grouped according to the International Classification of Diseases, 10th Revision (ICD‐10: I30–I52, I21), during the pre‐Omicron variant phase (September 2020 to December 2021; Table 2). The study found that full COVID‐19 vaccination was associated with a 51% reduction in the risk of these cardiovascular events at 28 days (Vaccinated: 253/33,836 vs. Unvaccinated: 543/33,836; RR = 0.49; 95% CI: 0.43–0.57) and a 65% reduction at 90 days (Vaccinated: 117/33,506 vs. Unvaccinated: 349/33,506; RR = 0.35; 95% CI: 0.29–0.44) [13].

**TABLE 2:** Identification of the studies included and main results.

Author (Year)/Country	Outcomes analysed and (period of execution)	Main results	
Zisis et al. (2022) [13]/USA	Post‐COVID‐19 heart disease at 28‐days and 90‐days (9/21/2020 to 12/14/2021)	**28‐Days** Vaccinated = 253/33,836 Unvaccinated = 543/33,836 RR (95% CI) = 0.49 (0.43–0.57) AR (95% CI) = −15.76 (−18.96 to 12.57) **90‐Days** Vaccinated = 117/33,506 Unvaccinated = 349/33,506 RR (95% CI) = 0.35 (0.29–0.44) AR (95% CI) = −13.07 (−15.55 to −10.60)	
Wan et al. (2023) [14]/China (Hong Kong)	Post‐COVID‐19 cardiovascular diseases (CVDs), coronary heart disease (CHD), heart failure (HF), after 28 days since COVID‐19 infection (Documented COVID‐19 infection between 2/23/2021 to 10/31/2022) End of the study (January 23, 2023)	**CVD** Unvaccinated = 6007/87,253; 1 dose = 5153/84,534; RR (95% CI) = 0.79 (0.71–0.88); 2 doses = 3951/425,965 RR (95% CI) = 0.63 (0.58–0.69) **CHD** Unvaccinated = 1906/87,253; 2 doses = 1391/425,965; RR (95% CI) = 0.70 (0.60–0.82) **HF** Unvaccinated = 1635/87,253; 1 dose = 1140/84,534; RR (95% CI) = 0.64 (0.52–0.78); 3 doses = 384/392,102; RR (95% CI) = 0.34 (0.27–0.43)	
Parodi, Indavere, Jacob, et al. (2023) [15]/Argentina	Post‐COVID cardiac injury (left ventricular ejection fraction (LVEF) ≤ 52% in men or ≤ 54% in women, regional wall motion abnormalities, or pericardial effusion) (9/1/2020 to 6/30/2022)	**Post‐COVID cardiac injury** Unvaccinated = 44/1070; Vaccinated = 11/883; OR (95% CI) = 0.32 (0.16–0.62) aOR (95% CI) = 0.33 (0.17–0.65)	
Kim, Maeda, Murata, Fukuda (2025) [16]/Japan	Post‐COVID conditions (Ischaemic heart disease, hypertension, pulmonary circulation disease, arrhythmia, heart failure and stroke) occurring within 8 months of COVID‐19, according to vaccination status. Distant group: COVID‐19 occurrence ≥ 365 days after vaccination. Intermediate group: COVID‐19 occurrence 150–364 days after vaccination. Recent group: COVID‐19 occurrence 14–149 days after vaccination. (8/2020 to 12/2022)	**Ischaemic heart disease** Distant group (66/26,179) Recent group (84/26,479) HR* = 0.55 (95% CI, 0.36–0.84) **Hypertension** Distant group (182/23,338) Recent group (206/16,257) HR* = 0.60 (95% CI, 0.45–0.79) **Pulmonary circulation disease** Distant group (09/27,565) Recent group (04/31,533) HR* = 0.09 (95% CI, 0.01–0.80)	**Arrhythmia** Distant group (89/26,573) Recent group (85/27,917) HR* = 0.57 (95% CI, 0.38–0.86) **Heart failure** Distant group (116/25,466) Recent group (137/24,516) HR* = 0.45 (95% CI, 0.32–0.63) **Stroke** Distant group (21/27,419) Recent group (26/31,132) HR* = 0.38 (95% CI, 0.16–0.89)
Polack, Thomas, Kitchin, et al. (2020) [17]/(Multicenter)	Efficacy of BNT162b2 (VE) against confirmed COVID‐19 at least 7 days after the second dose and VE against severe COVID‐19 occurrence after dose 1 (7/27/2020 to 11/14/2020)	**VE against confirmed COVID‐19** Unvaccinated = 162/18,325; Vaccinated = 08/18,198; VE = 95.0% (95% CI 90.3–97.6) **VE against severe COVID‐19** Unvaccinated = 09/21,259; Vaccinated = 01/21,314; VE = 88.9% (95% CI 20.1–99.7)	
Zheng, Shao, Chen, Zhang, Wang, Zhang (2021) [18]/Meta‐analysis (14 countries)	Vaccine effectiveness (VE) against SARS‐CoV‐2 infection, COVID‐19‐related hospitalisation, ICU admission/severe disease, and COVID‐19‐related death among fully vaccinated individuals (8/6/2020 to 10/6/2021)	**VE for the prevention of SARS‐CoV‐2 infection** 35 articles = 89.1% (95% CI 85.6%–92.6%) **VE against COVID‐19‐related hospitalisation** 15 articles = 97.2% (95% CI 96.1%–98.3%) **VE against COVID‐19‐related ICU admission or severe disease** 04 articles = 97.4% VE (95% CI 96.0%–98.8%) **VE against COVID‐19‐related death** 08 articles = 99.0% VE (95% CI 98.5%–99.6%)	
Janes, Fisher, Kee, et al. (2024) [19]/USA (Multicenter)	SARS‐CoV‐2 viral load (VL) (log10 copies/mL) at the time of COVID‐19 diagnosis, occurring ≥ 14 days (≥ 7 days for the novavax vaccine) after the last dose of the vaccine. (07/2020 to 07/2021)	Mean difference (VL) between vaccine versus placebo groups Moderna (36 vs. 594) −2.12 (−2.80 to −1.44) Novavax (11 vs. 60): −2.78 (−4.18 to −1.38)	
Drury, Camara, Chelysheva, et al. (2024) [20] ^/^ UK	Immune responses to SARS‐CoV‐2 virus in blood post‐vaccination with Oxford‐AstraZeneca COVID19 vaccine (ChAdOx1 nCoV‐19) or a placebo (Jun to Dec 2020)	Differences in serum cytokine concentrations observed at the onset of COVID‐19 between the placebo (*n* = 30) versus vaccines (*n* = 21). Higher levels of IFN‐γ, IL18, IP10, IL10 and TNF‐α in the placebo group in CT and CT+7, respectively: **IFN‐*γ* fdr = 0.042 and 0.**019; **IL18** fdr = 0.034; **IP10** fdr = 0.0035 and 0.0044; **IL10** fdr = 0.013 and 0.013; **TNF‐*α*** fdr = 0.0057 and 0.0057	
Behrouzi, Bhatt, Cannon, et al. (2022) [22]/Canada Meta‐analysis (06 studies)	Major adverse cardiovascular events related to the influenza virus (cardiovascular death or hospitalisation for myocardial infarction, unstable angina, stroke, heart failure, or urgent coronary revascularisation) within 12 months of follow‐up (2000–2021)	**Cardiovascular events related to the influenza virus** 3.6% (vaccinated = 162/4510) versus 5.4% (unvaccinated = 242/4491); RR = 0.66 (95% CI, 0.53–0.83; *p* < 0.001)	

Abbreviations: aOR, Adjusted Odds Ratio; AR, Attributable Risk; CT, COVID‐19 test visit; CT+7, Seven days later, Cytokines/chemokines—IFN‐*γ*, gamma interferon; HR*, hazard ratio adjusted for sex and age; ICU, Intensive Care Unit; IL10, Interleukin‐10; IL18, Interleukin‐18; IP10, Interferon‐gamma‐induced protein 10; OR, Odds Ratio; RR, Relative Risk; TNF‐*α*, tumour necrosis factor alpha; VE, Vaccine effectiveness.

The protective effect of vaccination was also assessed in a Chinese study. The authors examined reductions in the incidence of heart failure, stroke, and coronary artery disease following COVID‐19 infection, during the Omicron‐dominant wave period (February 2021 to October 2022; Table 2). In this study, a three‐dose vaccination regimen reduced the occurrence of these post‐COVID cardiovascular outcomes by 66% (Vaccinated 3 doses: 384/392,102 vs. Unvaccinated: 1635/87,253; RR = 0.34; 95% CI: 0.27–0.43) [14]. The quality of this evidence was evaluated in a systematic review, which rated the risk of bias in both studies as low [7].

### 4.2 Consistency

According to Hill, an association demonstrates consistency when it is observed repeatedly across different populations and under varying circumstances [11]. At the same time, consistency depends on the presence of complementary component causes required to complete a sufficient cause; therefore, the absence of consistency does not rule out a causal association [9].

The protective effect of COVID‐19 vaccination against post‐COVID cardiovascular conditions was observed in three cohort studies (*n* ≈ 1,050,000) conducted in Argentina [15], the United States [13], and China [14]. In all three settings, the results consistently demonstrated the protective effect of vaccination, with reductions in the risk of cardiovascular events. In Argentina, a 77% reduction in the risk of post‐COVID heart injury was observed (aOR = 0.33; 95% CI: 0.17–0.65; Table 2) [15].

### 4.3 Specificity

As proposed by Hill, specificity requires that a cause be responsible for a single effect [9]. Since vaccines primarily target the prevention of severe primary COVID‐19 infection, their protection against cardiovascular diseases as part of PCC would be considered a secondary effect. Furthermore, cardiovascular diseases have a complex causal chain with multiple determinants that extend beyond non‐modifiable biological factors, including the influence of environmental and interpersonal conditions [23]. Therefore, attributing a single effect to the association under consideration seems unwarranted.

A study conducted with U.S. veterans on the association between reinfections and the occurrence of PCC showed that, even among previously vaccinated individuals, the risk of post‐COVID cardiovascular disease was higher in those who experienced reinfection [24]. These findings support the view that infection or reinfection is a necessary, but not sufficient, causal factor for the development of these conditions, and that vaccination, while serving as an important protective factor, does not completely eliminate the risk. This aligns with the understanding that multiple factors contribute to the occurrence of both the infection and post‐COVID cardiovascular conditions. Thus, specificity, in this case, cannot be applied as a strict criterion.

The presence of other factors influences the causal relationship under study between vaccination and post‐COVID cardiovascular events, as highlighted by the SCC model. According to Hill, the absence of specificity does not rule out the possibility of a cause‐and‐effect relationship, since this criterion has limited utility for causal inference [10], particularly when studying diseases with a multicausal aetiology.

### 4.4 Temporality

Temporality is an indisputable criterion, referring to the requirement that a cause must occur before its effect [11].

Cohort studies conducted in the United States [13], Argentina [15], and Japan [16] provide robust evidence for establishing temporality between the analysed events (cause and effect). Across these three studies, it was demonstrated that exposure to the COVID‐19 vaccine precedes the evaluated post‐COVID cardiovascular conditions and is associated with a reduced risk of these outcomes (Table 2) [13, 15, 16]. Thus, the evidence satisfies the only indispensable criterion for causality according to both Hill and the SCC model, which stipulates that a component cause must necessarily precede the effect it generates [10].

### 4.5 Biological Gradient

This perspective, proposed by Hill, assumes that some associations exhibit a dose‐response relationship, with a gradual increase in effect as exposure to the causal agent increases [11]. However, a gradual increase in disease is not always observed with higher exposure levels [9].

Nevertheless, the relationship between vaccination and protection against post‐COVID cardiovascular conditions satisfies the biological gradient in the studies analysed. A cohort study conducted in China estimated that a single dose of the COVID‐19 vaccine conferred 36% protection against post‐COVID heart failure (RR = 0.64; 95% CI: 0.52–0.78), while three doses increased this protection to 66% (RR = 0.34; 95% CI: 0.27–0.43) [14].

A gradual increase in protection conferred by the COVID‐19 vaccine over time was also observed in the United States [13]. The study evaluated the vaccine's protective effect, administered prior to infection, on post‐COVID cardiovascular conditions, comparing outcomes at 28 days (RR = 0.49; 95% CI: 0.43–0.57) and 90 days after infection (RR = 0.35; 95% CI: 0.29–0.44), demonstrating a clear dose‐response relationship [13].

Additionally, a study conducted in Japan (August 2020 to December 2022) found greater protection against the occurrence of post‐COVID cardiovascular conditions in patients who contracted COVID‐19 within five months of vaccination compared to those infected 12 months or more after vaccination, suggesting that regular booster vaccinations may help reduce the risk of post‐COVID conditions. Hazard ratios (HRs) for the recent group (reference: distant group) of 0.45 (95% CI: 0.32–0.63; Recent: 137/24,516 vs. Distant: 116/25,466) for heart failure and 0.38 (95% CI: 0.16–0.89; Recent: 26/31,132 vs. Distant: 21/27,419) for stroke were observed (Table 2) [16].

### 4.6 Plausibility and Coherence

As the term suggests, plausibility implies the existence of a biologically plausible explanation for the proposed causal relationship. To some extent, plausibility depends on current knowledge [11] and prevailing scientific paradigms, and is thus not an absolute criterion [9].

Coherence, in turn, refers to the consistency of findings on the cause‐and‐effect relationship with existing biological knowledge and the natural history of the disease [11]. Knowledge about COVID‐19 is relatively recent, and the full biological mechanism explaining the vaccine's protective effect against post‐COVID cardiovascular conditions is not yet fully understood. Despite this, the explanatory hypotheses are plausible and consistent with current scientific understanding and with the natural history of COVID‐19.

The vaccine is known to be effective in preventing severe cases of the disease (Table 2) [17, 18]. Among the proposed mechanisms for its protective effect against post‐COVID cardiovascular conditions, it is believed that COVID‐19 vaccination reduces viral load, as demonstrated by randomized clinical trials (July 2020 to July 2021) (RCT) comparing vaccinated groups with placebo groups (36 vs. 594), showing a mean difference of −2.12 (−2.80 to −1.44) for Moderna and −2.78 (−4.18 to −1.38) for Novavax [19], and the proinflammatory responses associated with severe infection (Table 2) [20]. Consequently, this reduction in systemic inflammation mitigates myocardial damage and likely lowers the incidence of cardiovascular diseases in the post‐COVID period.

Figure 1 illustrates an example of the explanatory causal mechanism conceptualised within the framework of the sufficient‐component cause (SCC) model [9].

**FIGURE 1:**
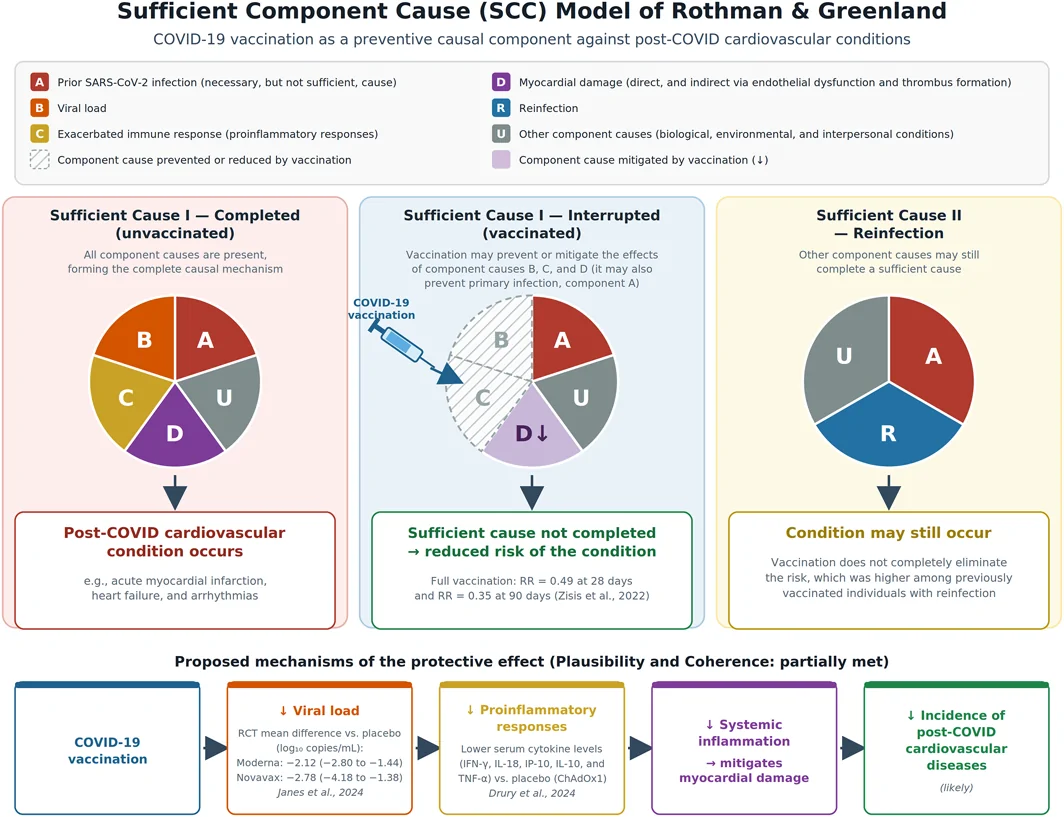
Application of the sufficient‐component cause model to the hypothetical causal pathways linking COVID‐19 vaccination to post‐COVID cardiovascular conditions. Each pie represents a sufficient cause, that is, a minimal set of component causes that inevitably produces the outcome [9]. Slice sizes are illustrative and do not indicate the relative contribution of each component. The proposed mechanisms are plausible but not yet fully understood.

### 4.7 Experimental Evidence

Although Hill considered experimental evidence as the strongest support for confirming a causal hypothesis [11], not every causal relationship can be easily demonstrated through experimental studies, which should be regarded as only one of several ways to test a causal hypothesis [9].

RCTs specifically addressing post‐COVID outcomes have not been identified in the literature. Nevertheless, several robust observational studies provide consistent evidence of an association between COVID‐19 vaccination and protection against cardiovascular outcomes beyond the acute phase of infection [13, 14, 15, 16, 25]. Ecological temporal relationships, such as increases in population‐level vaccine coverage accompanied by reductions in the incidence of post‐COVID cardiovascular conditions, could further support this link, although no studies specifically designed to test this hypothesis have yet been reported.

### 4.8 Analogy

Analogy implies that a causal relationship may exist if a comparable association is observed between the same outcome and a similar exposure, or between the same exposure and a similar outcome [10]. Hill argued that, in some situations, analogy can help infer a causal relationship [11]. However, this concept is among Hill's least robust criteria. Analogy can serve as a starting point for developing more robust hypotheses, but its absence does not necessarily rule out a causal hypothesis [9].

The COVID‐19 vaccine reduces the exacerbated immune response [20] that is associated with myocardial injury [21]. A similar mechanism has been observed with the influenza vaccine, which also lowers the occurrence of cardiovascular outcomes triggered by influenza infection (Cardiovascular events: Vaccinated = 162/4510 [3.6%] vs. Unvaccinated = 242/4491 [5.4%]; RR = 0.66; Table 2) [22]. Among other hypotheses, both vaccines appear to reduce systemic inflammatory processes that have been linked to the development of myocardial damage. However, the available studies did not specifically address persistent post‐infection cardiovascular complications in different vaccination contexts.

It is important to emphasise that the studies included in this analysis did not report variant‐specific SARS‐CoV‐2 infection rates. Given that different viral variants (ranging from the ancestral WA‐1 strain to subsequent lineages such as Delta and Omicron, which dominated the respective study periods) may be associated with distinct epidemiological and clinical risk profiles, comparisons of findings these studies should be interpreted with caution. Therefore, future studies should be designed to robustly stratify this risk and to follow patient cohorts longitudinally across subsequent and emerging variants, thereby ensuring accurate causal inferences across evolving epidemiological waves.

Table 2 presents the identification of the studies with the main results used to support the discussion on causality.

## 5 Conclusion

Focussing specifically on post‐COVID cardiovascular conditions, the scientific evidence indicates that COVID‐19 vaccination meets four BHC as a protective factor. Plausibility, coherence, and analogy are partially satisfied, whereas specificity and experimental evidence are not supported by the reviewed literature. The SCC model underscores the key role of vaccination as a preventive causal component. It also supports two BHC (strength of association and temporality), while highlighting the limitations of specificity in causal inference.

As noted by Hill and further emphasised by the SCC model, the specificity criterion is limited, and its absence does not refute the possibility of a causal relationship. Regarding experimental evidence, although no clinical trials specifically addressing post‐COVID cardiovascular outcomes were identified, the presence of robust observational studies among those cited indicates that the available evidence supports the potential cause‐and‐effect relationship under consideration.

The partially met criteria reflect the recent emergence of the disease and the existing gaps in understanding its biological mechanisms and the multiple factors involved, which influence both the occurrence of post‐COVID cardiovascular conditions and the vaccine's effect on cardiovascular outcomes in the post‐COVID context.

COVID‐19 vaccination has proven effective in preventing COVID‐19, particularly severe and fatal cases. Beyond its impact during the acute phase, evidence indicates that its protective effect also extends to reducing the risk of post‐COVID conditions. Strategies for complete immunisation and the prevention of reinfections are essential to mitigate the burden of post‐COVID cardiovascular conditions.

Additional studies are needed to explore the underlying biological mechanisms, potential interactions, and the role of COVID‐19 vaccination in preventing post‐COVID complications. Their results could inform public health policies and guide vaccination‐based prevention strategies.

## Author Contributions

L.F.B.S.M., E.C.D., I.P.S.F.d.C., and H.M.P. contributed to the conceptualisation and design of the study. L.F.B.S.M., E.C.D., and H.M.P. prepared the initial draft of the manuscript. M.C.X.N. provided specialised intellectual input regarding cardiology and medical expertise. All authors contributed to data analysis and interpretation, critically reviewed the manuscript for important intellectual content, and approved the final version for submission.

## Ethics Statement

The authors have nothing to report.

## Consent

The authors have nothing to report.

## Conflicts of Interest

The authors declare no conflicts of interest.

## Data Availability

Data sharing not applicable to this article as no datasets were generated or analysed during the current study.
